# Phospholipid exchange shows insulin receptor activity is supported by both the propensity to form wide bilayers and ordered raft domains

**DOI:** 10.1016/j.jbc.2021.101010

**Published:** 2021-07-26

**Authors:** Pavana Suresh, W. Todd Miller, Erwin London

**Affiliations:** 1 Department of Biochemistry and Cell Biology, Stony Brook University, Stony Brook, New York, USA; 2 Department of Physiology and Biophysics, Stony Brook University, Stony Brook, New York, USA; 3 Department of Veterans Affairs Medical Center, Northport, New York, USA

**Keywords:** autophosphorylation, bilayer width, cyclodextrin, insulin, insulin receptor, lipid exchange, liquid ordered, membrane lipid, membrane structure, lipid rafts, bSM, brain SM, CMC, critical micelle concentration, DDM, n-dodecyl-β-D maltopyranoside, DLPC, 1,2-dilauroyl-sn-glycero-3-phosphocholine, DM, n-decyl-β-D maltopyranoside, DMEM, Dulbecco’s modified eagle medium, DMPC, 1,2-dimyristoyl-sn-glycero-3-phosphocholine, DOPC, 1,2-dioleoyl-sn-glycero-3-phosphocholine, DPPC, 1,2-dipalmitoyl-sn-glycero-3-phosphocholine, DSPC, 1,2-distearoyl-sn-glycero-3-phosphocholine, eSM, egg SM, HDM, n-hexadecyl-β-D-maltopyranoside, IR, insulin receptor, Ld, liquid disordered, Lo, liquid ordered, MαCD, methyl-α-cyclodextrin, MβCD, methyl-β-cyclodextrin, PBS, phosphate buffered saline, PC, phosphatidylcholine, POPC, 1-palmitoyl-2-oleoyl-glycero-3-phosphocholine, SM, sphingomyelin, SOV, sodium orthovanadate, TDM, n-tetradecyl-β-D maltopyranoside

## Abstract

Insulin receptor (IR) is a membrane tyrosine kinase that mediates the response of cells to insulin. IR activity has been shown to be modulated by changes in plasma membrane lipid composition, but the properties and structural determinants of lipids mediating IR activity are poorly understood. Here, using efficient methyl-alpha-cyclodextrin mediated lipid exchange, we studied the effect of altering plasma membrane outer leaflet phospholipid composition upon the activity of IR in mammalian cells. After substitution of endogenous lipids with lipids having an ability to form liquid ordered (Lo) domains (sphingomyelins) or liquid disordered (Ld) domains (unsaturated phosphatidylcholines (PCs)), we found that the propensity of lipids to form ordered domains is required for high IR activity. Additional substitution experiments using a series of saturated PCs showed that IR activity increased substantially with increasing acyl chain length, which increases both bilayer width and the propensity to form ordered domains. Incorporating purified IR into alkyl maltoside micelles with increasing hydrocarbon lengths also increased IR activity, but more modestly than by increasing lipid acyl chain length in cells. These results suggest that the ability to form Lo domains as well as wide bilayer width contributes to increased IR activity. Inhibition of phosphatases showed that some of the lipid dependence of IR activity upon lipid structure reflected protection from phosphatases by lipids that support Lo domain formation. These results are consistent with a model in which a combination of bilayer width and ordered domain formation modulates IR activity *via* IR conformation and accessibility to phosphatases.

Signal transduction is thought to be influenced by lipid organization into domains that can facilitate receptor clustering (1). The two types of domains thought to exist in cells are liquid ordered domains (Lo) also known as lipid rafts and liquid disordered domains (Ld) (2). Lipid rafts are predominantly composed of saturated lipids such as sphingomyelin (SM), and cholesterol, with properties such as tight lipid packing, increased membrane thickness, and slower lateral diffusion rates relative to Ld domains, which are enriched in unsaturated phospholipids (3). Rafts have been associated in signaling by membrane growth factor receptors (4), immunoglobulin E receptors, and antigen receptors in T and B cells (5, 6, 7). Previous studies have reported that some receptors have preferential distribution in lipid rafts and/or that their activity responds to cellular cholesterol levels, which can alter domain formation and properties (4, 8).

Insulin receptor (IR) is a receptor tyrosine kinase involved in cellular glucose intake and lipid metabolism (9, 10). There is evidence associating IR activity with lipid rafts (11). Studies have reported a decrease in IR autophosphorylation activity, reduction in downstream signaling protein IRS-1, and a reduction in glucose uptake by cells after removal of raft-promoting cholesterol from cells (12, 13, 14), although it has the opposite effect in NPC1−/− mouse hepatocytes (15). However, cholesterol depletion has many effects upon membrane structure and cannot by itself demonstrate a functional role for lipid rafts (16, 17, 18). IR has also been shown to be sequestered in detergent-resistant membrane fractions after insulin stimulation (15, 19, 20), and in the related caveolae fractions (12, 13, 21). However, the relationship of detergent resistant membranes and caveolae to Lo domains in cells is complex (6, 22, 23). Thus, these observations are insufficient to definitively demonstrate an interaction of IR with lipid rafts *in vivo*, or that lipid rafts have a role in IR function (2, 24).

By using methyl-β-cyclodextrin (MβCD) to substitute cellular cholesterol with sterols having various propensities to aid Lo domain formation, we previously reported that IR autophosphorylation activity was only high in cells containing sterols having a propensity to form ordered domains (25). This modulation of IR activity could reflect one of several effects of sterol substitutions: altered ordered domain formation, altered membrane width, or altered lipid packing in a homogeneous membrane lacking domains. To investigate further, we performed methyl-α-cyclodextrin (MαCD)-catalysed outer leaflet lipid exchange on CHO cells stably expressing IR (CHO IR), replacing endogenous plasma membrane outer leaflet lipids with a series of phospholipids (including sphingomyelin) that support or disrupt rafts. We found that phospholipids with a propensity to form Lo domains resulted in activation of IR. This effect was partly due to the decreased accessibility of IR to phosphatases after substitution using phospholipids that promote Lo domain formation. In addition, we found that increased hydrocarbon width promoted IR activity. A model is proposed for how association of IR with Lo domains could control its activity.

## Results

### Lipid exchange is highly efficient and shows unaltered IR surface expression and minimal cell damage

**Figure 1:**
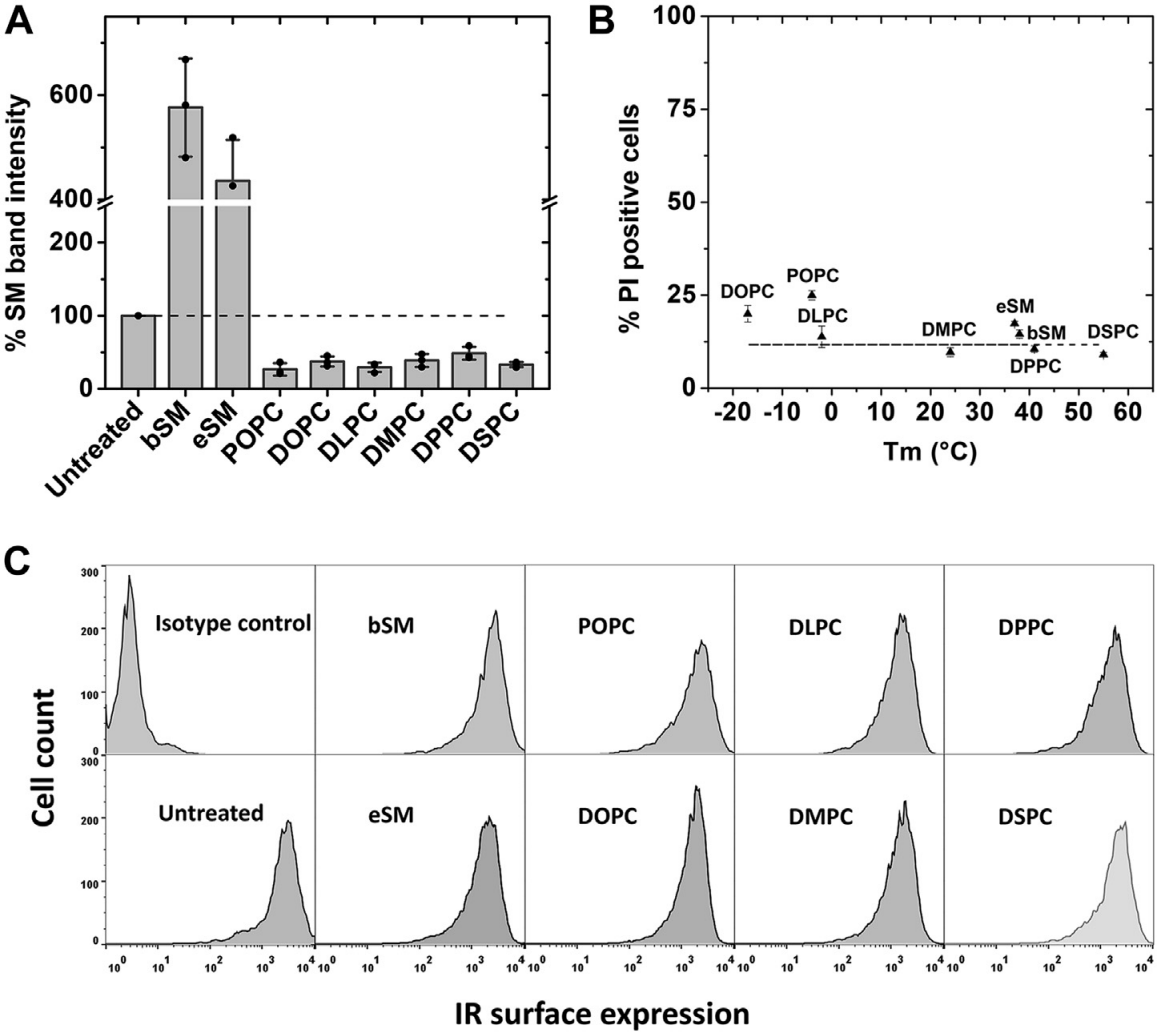
**CHO IR exchange efficiency, cell damage, and IR surface expression after lipid exchange.***A*, lipid exchange efficiency indicated by change in % SM band intensity compared with untreated cells. The average (mean) SM content ± S.D. and individual points from HP-TLC analysis of three independent experiments is shown. A representative HP-TLC image is shown in Fig. S1. All band intensities are shown normalized to untreated control SM levels set at 100%. A *dashed line* extends the 100% value for comparison to lipid exchange samples. *B*, cell damage monitored by propidium iodide (PI) staining using flow cytometry in lipid exchanged samples plotted *versus* the lipid gel-to-Ld phase transition temperatures (Tm). The average and S.D. from three experiments are shown. *C*, surface expression of IR using phycoerythrin conjugated IR antibody along with isotype control (nonbinding antibody).

We first carried out MαCD-catalyzed phospholipid exchange in CHO IR cells and analyzed the resulting change in SM content. Figure 1*A* shows exchange with SM increased the total SM content and exchange with phosphatidylcholines (PCs) decreased SM content. The efficiency of exchange by PCs was evaluated by comparing the loss of outer leaflet SM band in untreated cells. This can be used because replacement of endogenous lipid by exogenous lipid upon exchange is roughly 1:1 (26, 27). Exchange with all PCs (except 1,2-dipalmitoyl-sn-glycero-3-phosphocholine (DPPC)) showed ∼70% decrease of total SM in cells. This is close to the maximal level (∼70–80%) of endogenous cellular SM that can be removed by exchange, with the remaining SM likely being in a pool that is inaccessible to exchange (either being present in the inner leaflet of the plasma membrane or internal organelles) (26, 28). DPPC gave only 50% replacement of cellular SM, indicating somewhat lower efficiency of exchange. Based on this exchange data, we can infer that about 70% of total SM in the whole cell resides in the plasma membrane outer leaflet. Assuming exchange with exogenous SM is also highly efficient, (80–100%, as estimated in previous studies (26, 29)), the observed fivefold increase in SM would correspond to the plasma membrane phospholipid being 11 to 14% SM. This is in good agreement with the composition of SM content previously reported for CHO cell plasma membrane (28). Overall, exchange appears to be highly efficient, with the exogenous lipid forming the preponderance of phospholipid after exchange.

To ensure the cells were not damaged after lipid exchange, membrane permeability to propidium iodide was monitored using flow cytometry. The measurement was made after a 1 h incubation of lipid-exchanged cells in serum-containing media, as cells can be damaged by immediate processing after lipid exchange (29). Figure 1*B* shows that cell viability after exchange was similar to that in untreated cells. Similarly, IR surface expression was found to be largely unaltered immediately after lipid exchange as shown in Figure 1*C* by flow cytometry data using anti-IR phycoerythrin conjugated antibody raised against the IR ectodomain.

### Lipid exchange on CHO IR cells shows IR activity is supported in lipids that support ordered domains

**Figure 2:**
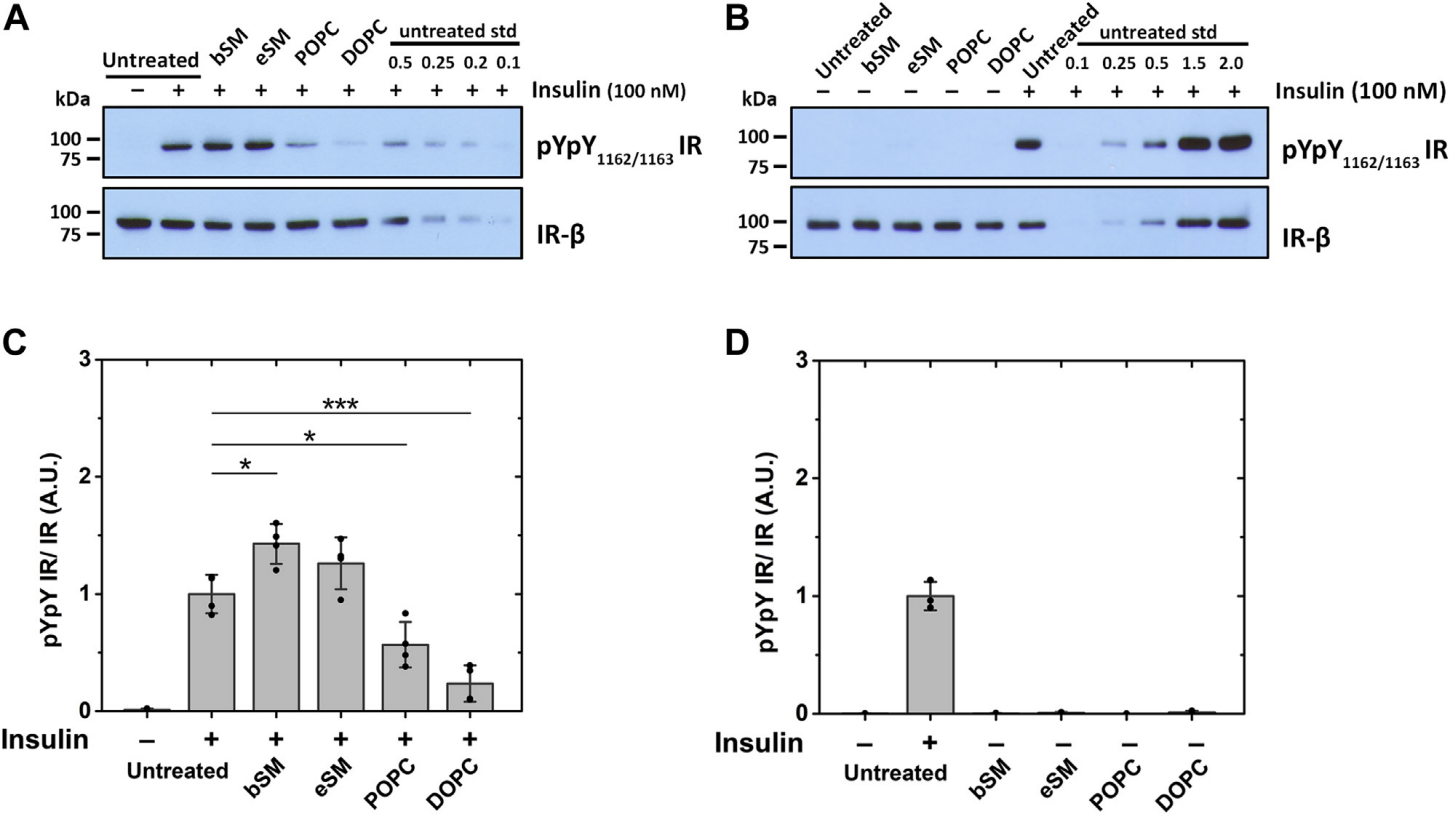
**IR autophosphorylation activity after lipid exchange on CHO IR cells with SMs and unsaturated PCs.** A representative western blot of (*A*) insulin stimulated cells and (*B*) unstimulated cells after lipid exchange with raft supporting bSM and eSM and nonraft supporting POPC and DOPC along with a standard curve of untreated insulin-stimulated control is shown. A standard curve with different loadings of the untreated sample (from 0.1× to up to 2×) is included on the blots. Quantification of western blot data is shown for (*C*) stimulated and (*D*) unstimulated cells after lipid exchange. Band intensities of pYpY IR were normalized to IR-β band intensity for each sample. In this and the following experiments, the average (mean), standard deviation, and individual experimental points are shown. Activity in insulin-stimulated untreated cells is defined as 1. ∗*p* < 0.05, ∗∗∗*p* < 0.001.

IR autophosphorylation activity was previously shown to be supported only by sterols that have a high propensity to form ordered domains in 293T IR cells (25). Similarly, in CHO IR cells we found that cholesterol depletion using MβCD decreased IR autophosphorylation activity and adding cholesterol back restored activity (data not shown). To further explore the effect of lipid structure on IR activity and its possible connection to Lo domain formation, we measured activity after MαCD-catalyzed outer leaflet plasma membrane lipid exchange in CHO IR cells with two different sphingomyelins (brain SM (bSM) and egg SM (eSM)), which have a propensity to form Lo domains, and with two unsaturated PCs (1-palmitoyl-2-oleoyl-glycero-3-phosphocholine (POPC) and 1,2-dioleoyl-sn-glycero-3-phosphocholine (DOPC)), which promote formation of the Ld state (30, 31) [Fig. 2]. Figure 2, *A* and *B* illustrate representative blots showing the effect of lipid exchange upon IR activity. To confirm western blot bands were within the linear range of detection, a standard curve of signal intensity *versus* protein was loaded on each blot [Fig. 2, *A* and *B* and Fig. S2]. Quantification of western blot data is shown in Figure 2, *C* and *D*. In subsequent figures, only the analyzed data is presented, but representative western blot images are shown in Fig. S3.

Relative to untreated cells, insulin-stimulated samples show slightly higher IR autophosphorylation activity after exchange with brain or egg SM and reduced activity after exchange with unsaturated PCs [Fig. 2*C*]. This reduction in IR autophosphorylation was most pronounced with DOPC, which is the lipid with the least ability to form ordered domains (30, 31, 32, 33). The correlation between IR activity and lipids that have a high propensity to form Lo domains is in agreement with our previous sterol substitution experiments (25). No significant autophosphorylation activity was observed in unstimulated samples with either SMs or PCs [Fig. 2*D*].

### Changing plasma membrane bilayer width using lipid exchange shows higher IR activity in wider membranes

The lower IR autophosphorylation activity seen in Ld domain supporting lipids could be a consequence of narrow membrane width in an Ld bilayer compared to an Lo bilayer. An Lo bilayer forms wider bilayers than Ld because of close packing of lipids, which minimizes gauche rotamers around C-C bonds (34). To investigate the role of membrane width in more detail, we carried out lipid exchange with saturated PCs having varying acyl chain lengths: di12:0 PC (1,2-dilauroyl-sn-glycero-3-phosphocholine (DLPC)), di14:0 PC (1,2-dimyristoyl-sn-glycero-3-phosphocholine (DMPC)), di16:0 PC (DPPC), and di18:0 PC (1,2-distearoyl-sn-glycero-3-phosphocholine (DSPC)). Since we are only changing the outer leaflet lipids, we expect a change in membrane thickness of ∼4.8 Å from DLPC to DSPC, assuming lengthening an acyl chain leads to a 0.8 Å increase in bilayer width per carbon atom (35). Estimated changes in plasma membrane widths for the Lo and Ld states after exchange with DLPC, DMPC, DPPC, and DSPC are shown in Table S1. These span a range from narrower to wider than on the unexchanged plasma membrane [Table S1]. In addition, experiments in asymmetric artificial lipid vesicles show that the formation of Lo domains increases monotonically as acyl chain length is increased, with DSPC forming the highest fraction of Lo domains (36).

**Figure 3:**
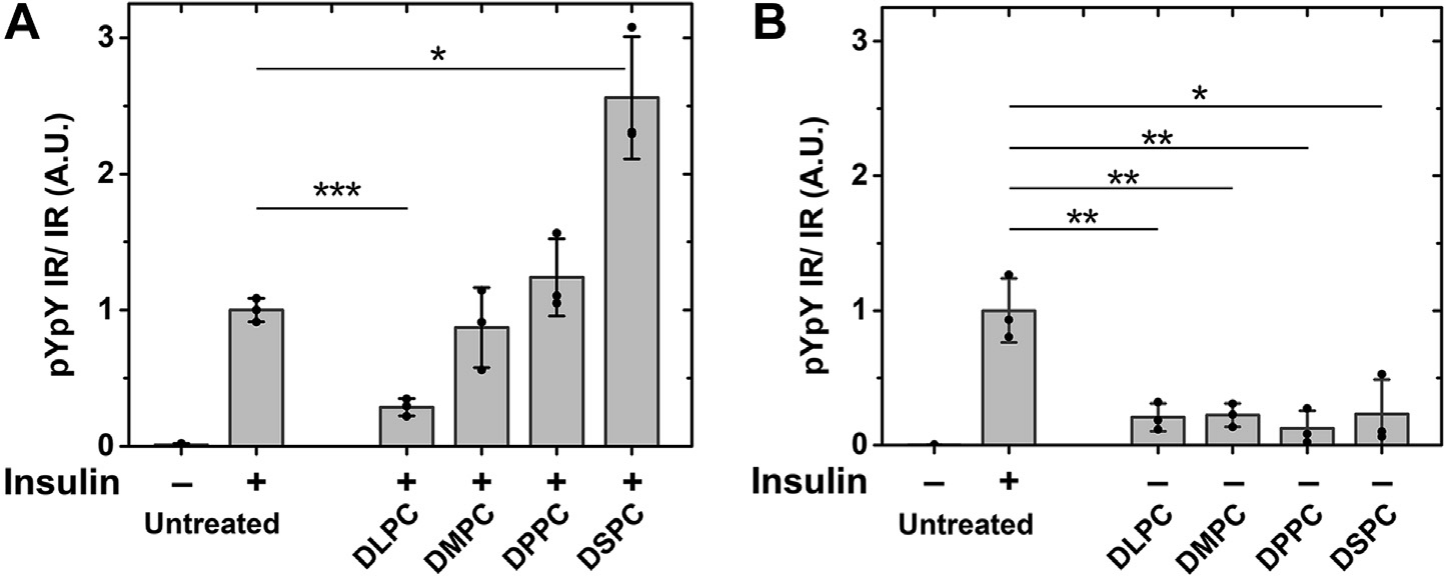
**IR autophosphorylation activity after lipid exchange with saturated PCs of short- and long-chain lipids.** Analysis of activity from western blot quantification of (*A*) stimulated and (*B*) unstimulated IR autophosphorylation activity after exchange with DLPC, DMPC, DPPC, and DSPC along with untreated control. ∗*p* < 0.05, ∗∗*p* < 0.01, ∗∗∗*p* < 0.001.

Figure 3*A* shows that in insulin-stimulated cells, IR autophosphorylation activity increased monotonically with acyl chain length. Compared with untreated cells, we observed a significantly lower IR activity with the shortest chain (DLPC), while the longest chain (DSPC) shows more than double the activity in untreated cells. After lipid exchange with saturated PCs, unstimulated cells showed a low level of basal autophosphorylation, but higher than the unstimulated untreated cells [Fig. 3*B*]. This suggests some loss of regulation of basal IR activity after introduction of saturated PCs.

### Strong correlation between propensity to form ordered state bilayers and increased IR activity

**Figure 4:**
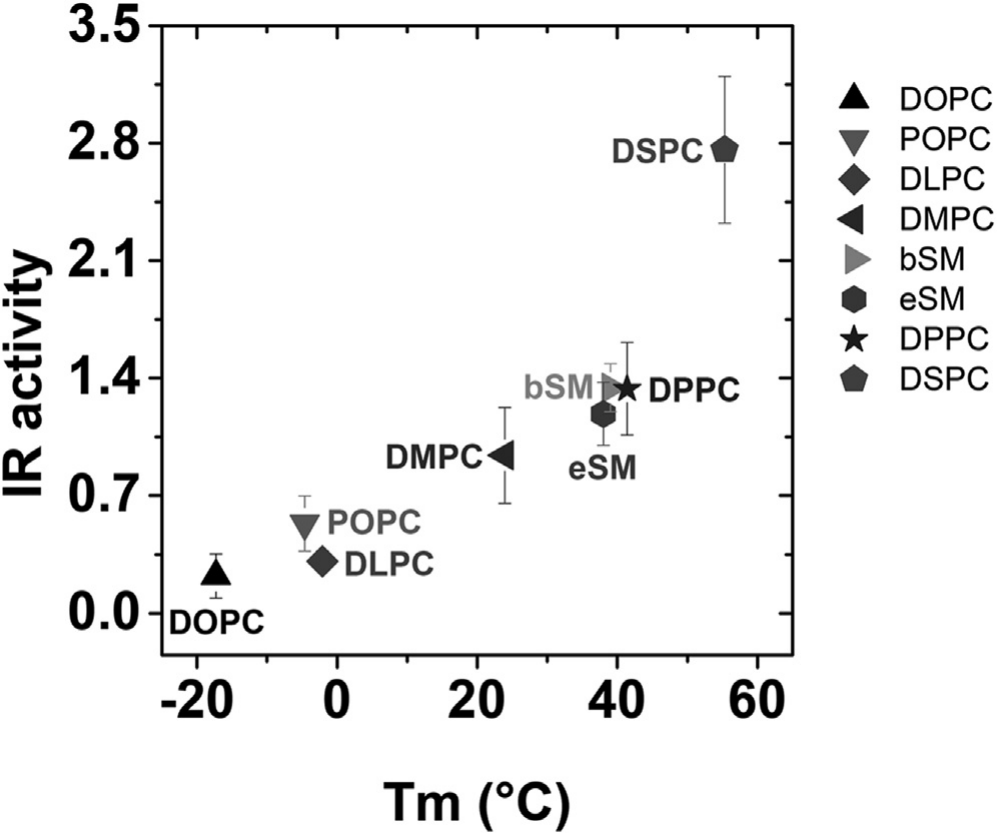
**Insulin stimulated IR autophosphorylation activity compiled from**Figures 2**and**3***versus* gel to Ld state lipid phase transition temperatures of exchanged lipid.** Activity in untreated cells has been normalized to a value of 1.

The observations above indicate that the effect of lipid exchange on the propensity of lipids to form the Lo state and its effect on bilayer width both regulate IR activity. Since saturated PCs that form wider bilayers have an increased propensity to form an ordered state, it is possible that the effect of lipid substitutions largely reflects degree to which lipid substitution enhances the ability to form the Lo state. A strong correlation between the ability to form an ordered state and IR activity supports this hypothesis. This correlation can be seen clearly in Figure 4 in which IR autophosphorylation activity is graphed against the gel-to-Ld phase transition temperatures (Tm) [Table S2] of the lipids in vesicles containing a single lipid species. The gel state is highly ordered, and in artifical asymmetric membranes high Tm is associated with a greater tendency to form Lo domains when cholesterol is present (36).

### IR activity in alkyl maltoside micelles of increasing micelle width shows higher IR activity in wider micelles

To test if there is also a direct effect of membrane width on IR activity, we measured the dependence of IR activity upon hydrophobic width under conditions in which there is no domain formation. This was done by incorporating purified full-length IR in alkyl maltoside micelles with increasing alkyl chain lengths (from 10 to 16 carbons). The detergents used were n-decyl-β-D maltopyranoside (DM), n-dodecyl-β-D maltopyranoside (DDM), n-tetradecyl-β-D maltopyranoside (TDM), and n-hexadecyl-β-D-maltopyranoside (HDM). Alkyl maltoside detergents are reported to form oblate ellipsoid structures with measured (or estimated) minor axis dimensions (widths) of 27 Å, 32 Å, 37 Å, and 41 Å for DM, DDM, TDM, and HDM, respectively (37) [Table S3]. For reference, the plasma membrane Ld and Lo thicknesses are approximately 33 Å and 37 Å, respectively (35), and for a homogeneous plasma membrane bilayer in which the outer leaflet is fully substituted with DLPC, DMPC, DPPC, or DSPC, we estimate bilayer widths of 30 to 39 Å [Table S1] depending on the physical state of the membrane. Thus, the detergent micelles roughly span the hydrophobic widths examined in saturated PC exchange experiments.

To carry out the experiments in detergent, IR was first affinity purified in DDM lysis buffer at 0.29 mM (critical micelle concentration (CMC) of DDM is 0.17 mM). The IR dissolved in DDM was diluted 1:10 with each of the four detergents, which were at concentrations of (0.5 + CMC) mM, to ensure 0.5 mM of detergent would be in the form of micelles in all cases [Table S3]. The DDM present after dilution should not significantly alter the average alkyl chain length. Even if all the diluted DDM is incorporated into the micelles of DM, TDM, and HDM, it would alter hydrophobic width by less than 0.5 Å.

**Figure 5:**
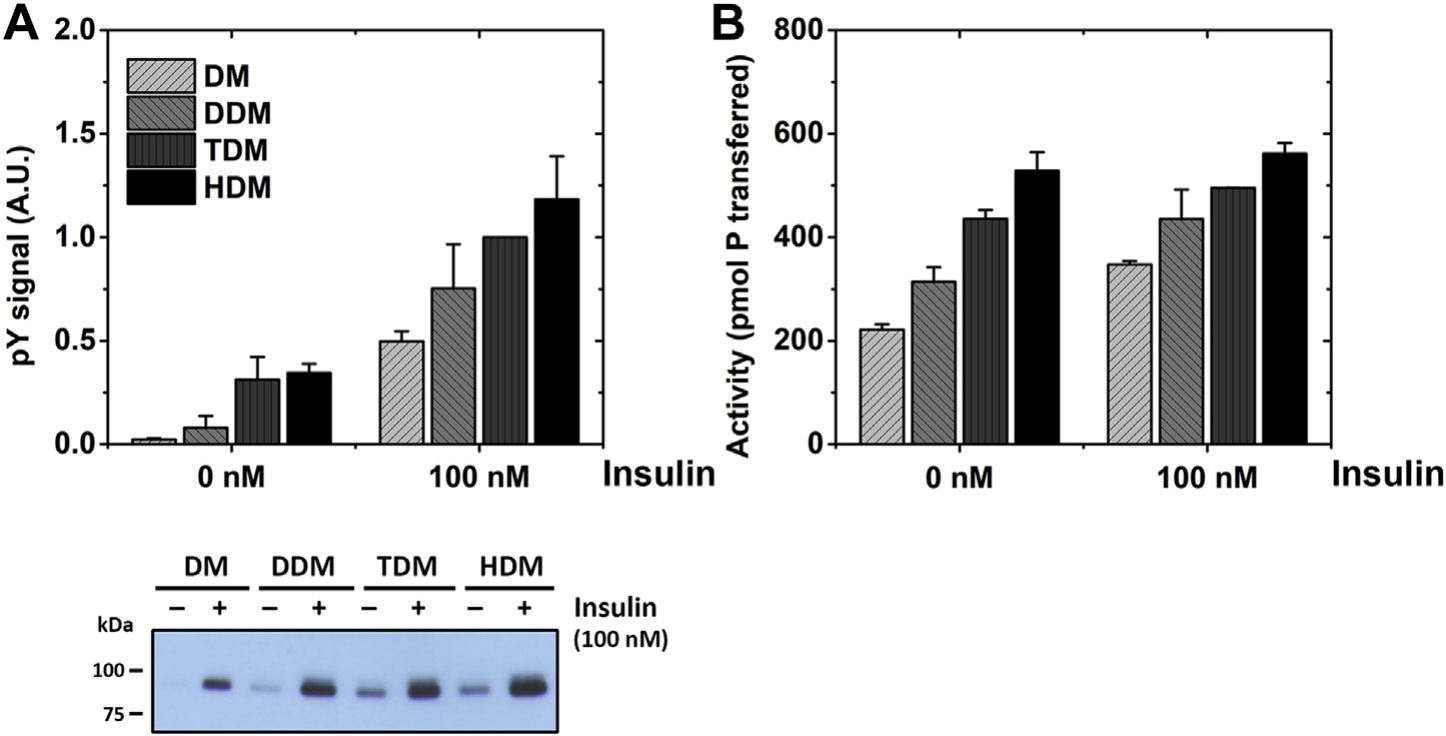
**IR kinase activity increases in maltoside micelles of increasing micelle width.***A*, IR autophosphorylation activity measured in maltoside micelles by western blot using phosphotyrosine antibody with and without 100 nM insulin. Densitometry analysis of bands from three independent experiments normalized to “100 nM insulin TDM” sample in each blot is shown. A representative sample blot is depicted below. *p*-values for difference of mean values of DM and HDM are *p* < 0.001 at 0 nM insulin and *p* < 0.01 at 100 nM insulin. *B*, radioactive kinase assay of IR activity on peptide substrate E_4_YM_4_ with and without 100 nM insulin stimulation measured as pico-mol phosphate transferred to peptide from three independent experiments is shown. *p*-values for difference of mean values of DM and HDM samples are *p* < 0.001 for both 0 nM and 100 nM insulin.

Insulin-stimulated IR autophosphorylation activity measured by western blotting with an antiphosphotyrosine antibody showed an increase with increasing micelle width [Fig. 5*A*]. The difference between the narrowest and widest micelles was about twofold. This is much less than the change in activity observed when membrane width was altered by lipid exchange. For wider micelles, the activity increase was also seen in samples lacking insulin stimulation [Fig. 5*A*].

We also assayed ATP-dependent IR-catalyzed phosphorylation of the synthetic peptide substrate E_4_YM_4_ (KKEEEEYMMMMG) in the alkyl maltoside micelles [Fig. 5*B*]. The activity of the receptor toward peptide substrate showed the same trend of increasing activity with increasing micelle width, but the difference between the thinnest and widest micelles was only 1.5-fold. In this assay, activity was only slightly higher in the presence of insulin relative to that in its absence. Thus, both the autophosphorylation and peptide phosphorylation data indicate that the formation of a substantial level of the active IR conformation does not require insulin binding in detergent micelles.

### Global inhibition of phosphotyrosine phosphatases shows phosphatases are partially responsible for the change in IR activity observed after lipid exchanges

The above experiments indicate that IR is considerably activated when membranes have a greater propensity to form Lo domains, but an increase in membrane width does not fully explain the large increase in IR activity. We next examined if a lipid-dependent decrease in accessibility to phosphatases was involved. Phosphorylation levels in cells involve a dynamic interplay between kinases and phosphatases (38, 39). For other receptor kinases believed to be activated by localization in Lo domains, segregation from phosphatases located in Ld domains is thought to be an important factor in enhancement of kinase activity (40, 41). If IR localization in Lo domains has a similar effect on accessibility to phosphatases, it would be predicted that phosphatases have less effect when the lipid substitutions increase the propensity to form Lo domains.

**Figure 6:**
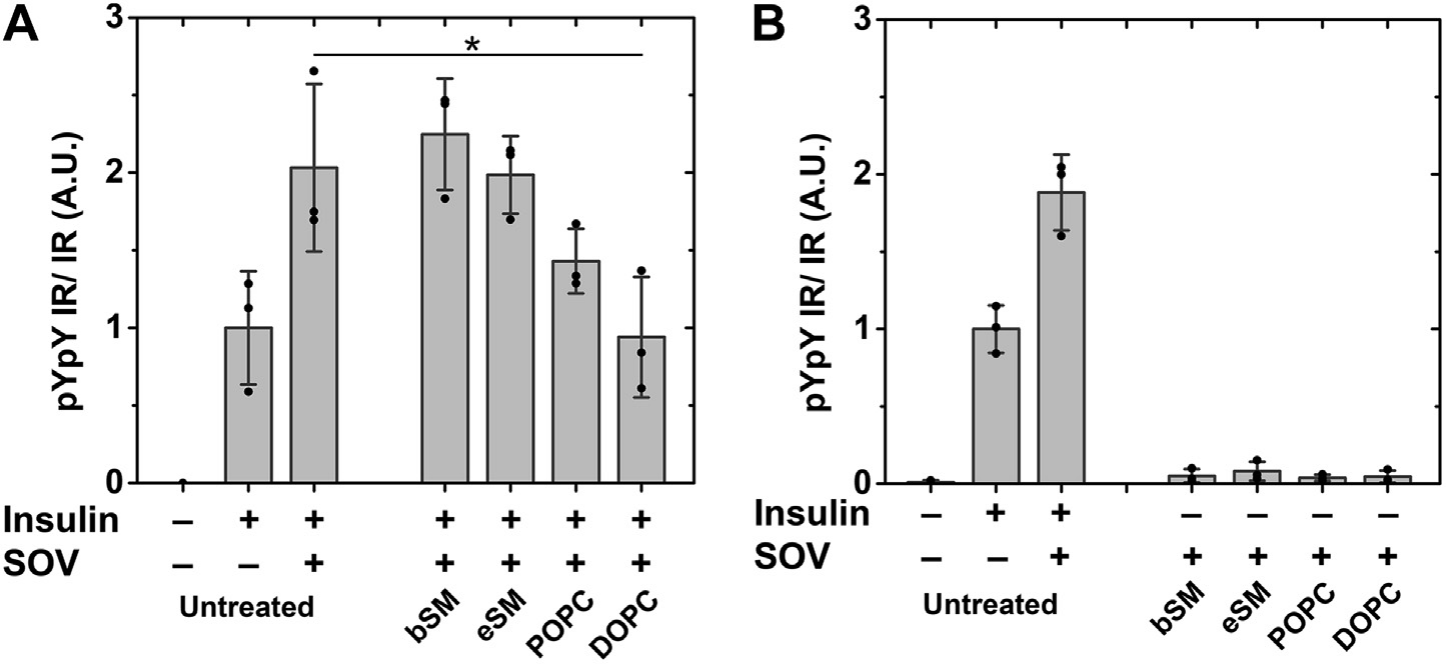
**IR activity in the presence of SOV after lipid exchange using SMs and unsaturated PCs.** Activity of insulin-stimulated (*A*) and unstimulated (*B*) cells after lipid exchange is shown. ∗*p* < 0.05.

To examine the effect of phosphatases on autophosphorylation, we compared activity after lipid exchange experiments in the absence and presence of a global phosphotyrosine phosphatase inhibitor, sodium orthovanadate (SOV) (42). Figure 6*A* shows a partial rescue of insulin-stimulated IR autophosphorylation activity after POPC and DOPC exchange in the presence of SOV. However, the IR activity was not fully restored in POPC and DOPC when compared with the SOV-treated control (untreated by lipid exchange) and to the SM exchanged samples. It is not clear whether this reflects lower intrinsic kinase activity or incomplete inhibition of phosphatases. Unstimulated samples showed no significant activity after lipid exchange when SOV was present [Fig. 6*B*].

**Figure 7:**
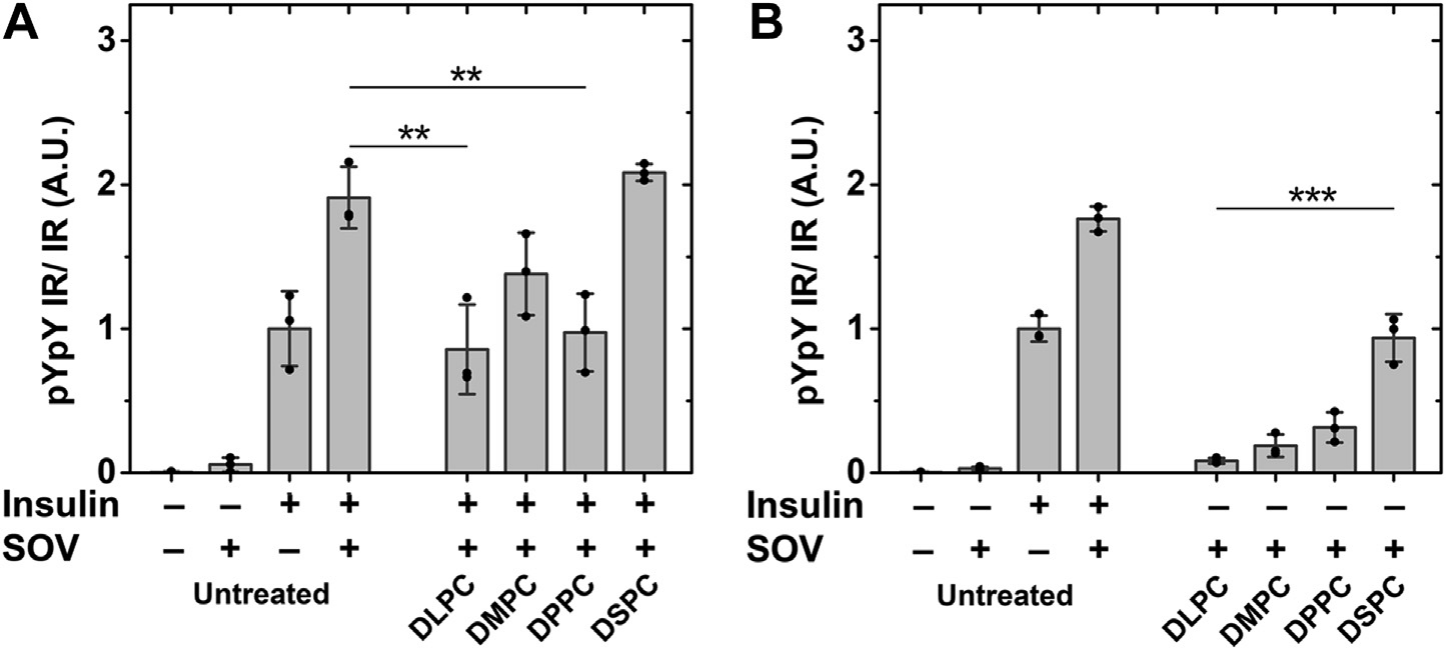
**IR activity in the presence of SOV after lipid exchange with saturated PCs of different chain lengths.** Activity of insulin-stimulated (*A*) and unstimulated cells (*B*) after treatment with SOV is shown. ∗∗*p* < 0.01, ∗∗∗*p* < 0.05.

There was also an effect of global phosphatase inhibition using SOV on cells that had undergone substitution with various saturated PCs. Figure 7*A* shows that after insulin stimulation in SOV-treated cells, the effect of acyl chain length upon activity was much smaller than in the absence of SOV (see Fig. 3).

Interestingly, in the presence of SOV, there was an increase in unstimulated IR activity with increasing PC acyl chain length [Fig. 7*B*]. DSPC exchanged cells in the absence of insulin had an activity similar to that in unexchanged insulin-stimulated cells. This shows that a very wide membrane is sufficient to activate IR in the absence of insulin.

**Figure 8:**
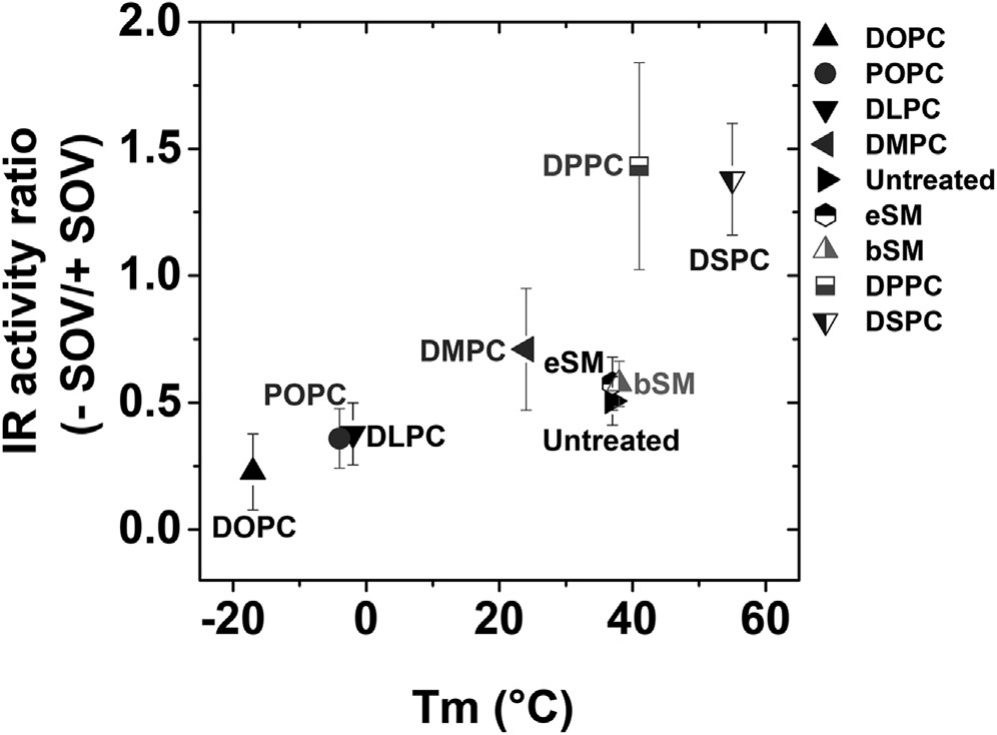
**Correlation of relative effect of phosphatases on IR activity with gel-to-Ld Tm phase transition temperature of exchanged lipid.** IR autophosphorylation activity without SOV is divided by IR activity with SOV treatment to illustrate the relative effect of phosphatases. A low value indicates a high degree of accessibility to phosphatases. The untreated sample is shown arbitrarily at a Tm of 37 °C.

To better illustrate the effect of phosphatases on IR activity, we graphed the relative effect of phosphatases (IR activity without SOV divided by IR activity with SOV) *versus* Tm [Fig. 8]. A value of 1 indicates no significant difference in IR activity in the presence (–SOV) or absence of phosphatase activity (+SOV), while a value below 1 suggests a higher IR activity in the absence of phosphatase activity (+SOV) indicating that phosphatases have a substantial role in removing the activity of IR after exchange. Low Tm lipids such as DOPC, DLPC, and POPC that have a lower propensity to form Lo domains have values below 0.5 showing the greatest effect after phosphatase inhibition [Fig. 8]. This might indicate a higher accessibility of phosphatases to IR in Ld domains.

Since IR activity was measured at room temperature, about 23 °C, it is possible that the observed effect of lipid domains on IR activity was more pronounced than at physiological temperature because lipid Lo domain formation is favored at lower temperatures (43). To test this, activity and subsequent processing were measured at 37 °C. We carried out these experiments on untreated cells and using as representative lipids DOPC (lipid with least propensity to form Lo domains) and DSPC (lipid with the highest propensity to form Lo domains). Similar to the results shown in Figures 2 and 3, IR autophosphorylation activity was low after DOPC exchange and high after DSPC exchange, compared with the untreated control (Fig. S4). Treatment with SOV showed that at room temperature and at 37 °C, the largest increase in activity was measured in DOPC-exchanged samples, intermediate increase in activity in untreated samples, and no increase in activity in DSPC-exchanged samples (Fig. S4). These results suggest that the observed IR activity responses to lipid exchanges are relevant at physiological temperatures.

## Discussion

Our previous study using sterol replacement after cholesterol depletion suggested that sterols supporting ordered domains provide a favorable environment for IR to be activated (25). In this study, the role of lipid organization on IR autophosphorylation activity in cells was investigated by manipulating the plasma membrane outer leaflet using methyl-α-cyclodextrin (MαCD)-mediated lipid exchange (26, 29). In agreement with the sterol results, insulin-stimulated IR autophosphorylation activity is higher in plasma membranes exchanged with lipids that have a higher propensity to form wide bilayers and Lo domains (high Tm lipids). We also observed a direct membrane thickness effect on IR activity, with wider maltoside micelle hydrophobic widths supporting higher activity of purified IR. Combined with the fact that Lo domains are wider than Ld domains, these experiments are consistent with the model that increased association of IR with Lo domains is responsible for the increased IR activity in lipids that tend to support Lo domains.

**Figure 9:**
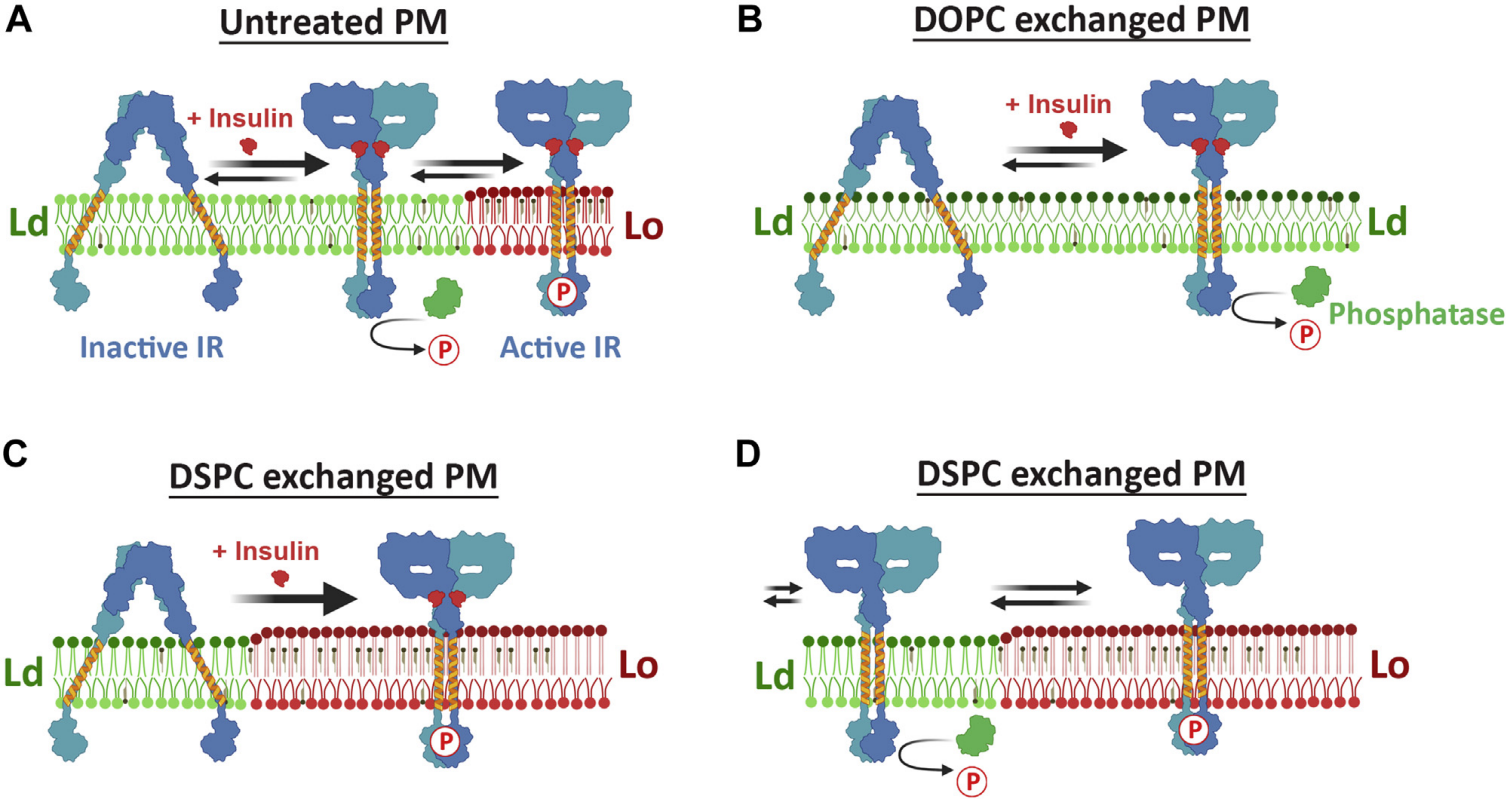
**Schematic representation of the hypothesis for the effect of altering lipid composition upon IR activity.***Green* represents Ld domains, *red* Lo domains. Phosphate is shown as P. Note: inactive IR is shown with tilted TM domains, while the active has an untilted conformation. *A*, in normal (untreated) plasma membrane, insulin binding promotes a conformational change that brings the kinase domains close together. This active conformation is supported in Lo domains where IR is protected from phosphatases and autophosphorylation proceeds. In Ld domains, phosphatases suppress the IR autophosphorylation. *B*, in DOPC exchanged membranes with largely Ld environment, IR is not protected from phosphatases, resulting in reduced autophosphorylation. *C*, in DSPC-exchanged membranes, which are largely Lo domain forming with wider membranes, the untilted active conformation of insulin stimulated IR is favored and strongly localized in Lo domains and therefore protected from phosphatase activity. *D*, even in the absence of insulin in DSPC-exchanged membranes, IR can form its active conformation and dynamically move between the Lo and Ld domains, with IR inactivated by phosphatases when it moves to the Ld domain. In *C* and *D*, the active and inactive IR conformations co-exist in the Ld domains, but only one conformation is shown for clarity.

An alternative model is that before and after exchange, the plasma membrane remains homogeneous, lacking domains, and that the effect of activating IR after lipid exchange is due to an increase in bilayer width. This alternative model is less likely to be correct for several reasons. First, the activating effect of increasing PC hydrocarbon chain length is much more than the effect of changing detergent hydrocarbon chain length. If there are no domains, one would predict they should have had similar levels of activation. Second, studies in plasma membrane vesicles formed after lipid exchange and in artificial asymmetric lipid vesicles show that lipid compositions increasing the propensity to form Lo domains do result in increased formation of ordered lipid domains under physiologic or near physiologic conditions (36, 43). Third, the domain model is favored by the close relationship between decreased sensitivity of IR activity to phosphatases in lipid substitutions that favor Lo domain formation. This suggests a sequestration where IR in Lo domains is protected from phosphatases [Fig. 9]. It has been shown in other signal transduction systems that activation of receptor complexes with kinase activity is enhanced by segregation of phosphatases in Ld domains and kinases in Lo domains. For example, activated Lyn kinase is sequestered in ordered Lo domains, where it is protected from the action of phosphatases, which reside in Ld domains (40). The main phosphatase known to interact and dephosphorylate IR is PTP1B (44, 45), an endoplasmic reticulum targeted protein tyrosine phosphatase, which has shown to be present in both caveolin associated rafts and nonrafts based on sucrose density fractionation (46, 47). While the distribution of PTP1B does not show a preferential partitioning to lipid rafts, its localization after insulin stimulation has not been studied. It is also possible that other plasma membrane phosphatases (44) are involved. In addition to, or instead of changing the proximity of IR and phosphatases, IR in an Lo state bilayer may exist in a conformation in which phosphatases are unable to act upon it.

In this regard, the behavior of IR after substitution with DSPC is especially noteworthy. After DSPC substitution, which should increase bilayer width to a value larger than in unexchanged cells, the activity of IR is higher than in unexchanged cells. Furthermore, there is no effect of inhibiting phosphatases on insulin-stimulated IR activity after DSPC exchange. This is consistent with a model in which insulin activated IR is totally sequestered in Lo domains after DSPC substitution [Fig. 9*C*]. In hepatocytes, IR being recruited into lipid rafts after insulin stimulation has been inferred from localization in raft-related detergent-resistant membrane (DRM) fractions (15, 19, 20). After insulin stimulation, autophosphorylated IR is predominantly seen in DRM fractions. Even in the absence of insulin stimulation, IR is very active after DSPC exchange when phosphatases are inhibited. This suggests a scenario where unstimulated IR dynamically shifts between Lo and Ld domains, and IR phosphorylation is removed by phosphatases when it is in Ld domains. Insulin addition likely helps to lock the active conformation of IR and thus allows for IR to be in Lo domains longer for increased signal transduction.

Our results can also be partly explained by the effect of membrane width on IR activity. In this regard, it should be noted that results from other groups also suggest IR activity can be influenced by membrane width. For example, in ceramide synthase 2 null mice hepatocytes, there is a reduction in ceramide acyl chain length and a loss of IR activity (20). This was attributed to a lesser IR localization in DRM but could also be partly due to reduced bilayer width. Additionally, in hepatocytes from mice with Niemann-pick type C disease, fatty acid chain lengths and IR activity were reduced (15).

The activation of IR in wider membranes could be a consequence of a change in IR transmembrane (TM) helix orientation within the membrane in the active form *versus* inactive form. IR in its inactivated conformation takes on an “inverted V-shape” form (48), which would be tilted relative to the plane of the membrane [Fig. 9], and therefore might favor formation of a tilted TM helix (48). The active “T-shaped dimer” conformation, which would be untilted with respect to the plane of the membrane, would be more likely to favor a TM helix that is not tilted. The TM helix of IR is about 23 residues long, so it could span the hydrophobic core of a bilayer of 34.5 Å without tilting. In narrower membranes, one would expect IR TM helices to be tilted to fit the hydrophobic width of the bilayer. In wider membranes such as DSPC (estimated 38.6 Å in the Lo state), the untilted TM helix would be strongly favored (perhaps with some accompanying local narrowing of the bilayer) (49). Thus, in a wider bilayer the untilted helices could tend to favor the T-shaped dimer, especially if the connection between extracellular and TM domains is very rigid. This could also explain activation of IR even in the absence of insulin after DSPC exchange, which would result in the widest bilayer width. Consistent with this model, it has been shown in previous studies that long TM segment length can favor a stronger affinity of TM proteins for Lo domains (50, 51), and this is due to the wider bilayer in the Lo domains (50). This model is illustrated in Figure 9. Thus, the results of plasma membrane lipid exchange studies in cells strongly favor a model of IR activity regulation where a combination of increased bilayer width and a relative inaccessibility of phosphatases in Lo domains favors IR activation. This model for kinase regulation may also be applicable to other signal transduction processes in which kinases have a single TM helix.

## Experimental procedures

### Materials

Studies used previously generated Chinese hamster ovary cells stably expressing human IR (CHO IR cells) (52). 293T cells stably expressing human IR (293T IR cells) with a C terminal streptavidin-binding peptide (SBP) tag were generated previously (25, 53). bSM, eSM, POPC, DOPC, DLPC, DMPC, DPPC, DSPC, and cholesterol were from Avanti Polar Lipids. MαCD was purchased from AraChem. Methotrexate and sodium orthovanadate (SOV) were purchased from Sigma Aldrich. Dulbecco’s modified eagle medium (DMEM, 4.5 g/l glucose, L-glutamine, sodium pyruvate), phosphate buffered saline (PBS) without calcium and magnesium (0.144 g/l KH_2_PO_4_, 9 g/l NaCl, 0.795 g/l Na_2_HPO_4_ (anhydrous)), trypsin-EDTA, antibiotic-antimycotic solution, and L-glutamine were purchased from Corning. Nonessential amino acids and ham’s F12 media were purchased from Gibco. G418 (geneticin) was from Goldbio, fetal bovine serum (FBS) was from VWR international, TDM and HDM were from Anatrace. DM was from Dojindo Molecular Technologies, DDM was from Thermo Fisher.

### Lipid and cholesterol purity

Lipid stock solutions in chloroform were checked for purity by chromatographing them on a high-performance thin-layer chromatography (HP-TLC) in 65:35:5 (v:v:v) chloroform:methanol:28.0 to 30.0 (v/v) % ammonium hydroxide. All lipids were present as single bands except eSM and bSM, which have a mixture of acyl chain lengths and thus show multiple bands. Cholesterol stock solution was similarly checked for purity in 3:2 (v:v) hexanes:ethyl acetate and a single band was observed.

### Cell culture

CHO IR cells were grown in DMEM supplemented with 10% FBS, 300 μg/ml glutamine, 100 μg/ml nonessential amino acids, 50 μg/ml G418, 2 μM methotrexate, and antibiotic–antimycotic solution (diluting 100× stock into medium). 293T IR cells were cultured in DMEM 4.5 g/l glucose media supplemented with 10% FBS and antibiotic–antimycotic solution. All cells were grown in a 37 °C incubator with 5% CO_2_. CHO IR cells were starved in serum-free ham’s F12 media.

### Preparation of lipid exchange media

Lipid exchange media with lipid-loaded MαCD was prepared as described previously (26, 29). Briefly, the desired amounts of lipids in chloroform were dried under N_2_ gas stream and further dried under high vacuum for 1 h. Multilamellar vesicles (MLVs) were formed by adding serum-free ham’s F12 media to dried lipids and incubating in a 70 °C water bath for 5 min followed by vortexing to disperse the lipid. MLVs and MαCD (from a stock of 300–400 mM in PBS) were added to serum free ham’s F12 media to prepare lipid exchange mixtures with the desired final concentrations. These were solutions with 40 mM MαCD and 0.5 mM lipid for DSPC, 1 mM lipid for bSM, eSM, and DPPC, 2 mM lipid for DMPC, and 4 mM lipid for POPC, DOPC, and DLPC. All lipid exchange mixtures except for DPPC and DSPC were incubated in 37 °C water bath for 30 min to allow lipids to be loaded onto MαCD and incubated in room temperature for 20 min before adding to cells. DPPC and DSPC exchange mixtures were incubated in 70 °C water bath for 30 min and then placed in room temperature for 30 min before adding to cells.

### Lipid exchange in CHO IR cells

Cells were grown to 80 to 90% confluency in 60 mm plates and lipid exchange was performed as described (29). Briefly, cells were washed in PBS twice at room temperature and starved in serum-free media for 22 h at 37 °C in a 5% CO_2_ incubator. The cells were then washed in PBS once and 1 ml lipid exchange mixture was added. Exchange was carried out at 26 to 27 °C for 1 h in an incubator without CO_2_. After exchange, cells were washed in PBS thrice.

### IR autophosphorylation

For measurement of IR autophosphorylation at room temperature, after lipid exchange, CHO IR cells in 60 mm plates were incubated with or without 100 nM insulin (in serum-free ham’s F-12 media) for 5 min at room temperature. Stimulation was terminated by removing insulin-containing media and adding 1 ml cold PBS after a 1 ml cold PBS wash. Cells were scraped from the plate and pelleted by centrifugation for 5 min at 1000*g* (at 4 °C) and lysed with 150 to 200 μl lysis buffer (50 mM Tris pH 8.0, 200 mM NaCl, 1% (v/v) Triton X-100, 1 mM EDTA, 1% (w/v) sodium deoxycholate, 10 μg/ml leupeptin, 10 μg/ml aprotinin, and 1 mM activated sodium orthovanadate). Lysates were centrifuged at 16,837*g* for 10 min (at 4 °C), aliquots were removed and then mixed with 5× Laemmli buffer (54) for western blotting analysis of autophosphorylation and protein levels. The remaining lysates were used to determine protein concentration using the Bradford assay (55).

For measurement of IR autophosphorylation at 37 °C after 1 h of lipid exchange in 60 mm plates, cells were washed in warm PBS (prewarmed at 37 °C) thrice. Then 100 nM insulin in serum-free ham’s F-12 media (prewarmed at 37 °C) was added, and cells were incubated in 37 °C, 5% CO_2_ incubator for 5 min. After insulin stimulation, insulin was removed, and cells were washed once in warm PBS (prewarmed at 37 °C). Lysis buffer was added directly on plate and cell lysates were collected by scraping. Lysates were centrifuged at 16,837*g* for 10 min (at RT), aliquots were removed and then mixed with 5× Laemmli buffer for western blotting analysis of autophosphorylation and protein levels. The remaining lysates were used to determine protein concentration using the Bradford assay.

### Western blotting

Whole cell lysate samples (typically 5–10 μg total protein) were loaded in equal amounts across all lanes (based on protein concentration using Bradford assay) and run on 7.5% acrylamide SDS-PAGE and then transferred to polyvinylidene fluoride (PVDF) membranes (Millipore) for 1 h at 100 V at 4 °C. Membranes were blocked with 5% (w/v) BSA in Tris-Buffered Saline and Tween 20 (TBST, 20 mM Tris, 137 mM NaCl, and 0.1% v/v Tween 20, pH 7.6) for 1 h and then incubated with primary antibodies overnight at 4 °C, followed by secondary antibodies for 30 min at room temperature. Membranes were imaged using western blotting substrate (Thermo scientific pierce ECL) and exposed to autoradiographic film (Bioexcell). Primary antibodies used were Anti-pYpY1162/1163 IR (Catalog number AF2507, R&D systems Inc), anti-insulin receptor β (Catalog number 3025, Cell signaling technology), and anti-phosphotyrosine (Catalog number 05-321, Millipore). Secondary antibodies used were rabbit IgG HRP conjugated (GE Healthcare life sciences) or mouse IgG HRP conjugated (GE Healthcare life sciences). All primary antibodies were diluted 1:1000 in TBST with 5% BSA from the commercially provided solution, except pYpY1162/1163 IR, which was used at 1:400 dilution in TBST with 5% BSA. Secondary antibodies were diluted to 1:5000 in TBST. To avoid having to strip membranes, duplicate gels were run, one for probing with pYpY1162/1163 IR and one for IR-β.

### Sodium orthovanadate (SOV) treatment with lipid exchange

Lipid exchange was carried out as described (see above) with activated SOV (42) added prior to exchange to a concentration of 1 mM in 1 ml of exchange media. For untreated samples, SOV was added at a concentration of 1 mM in 1 ml serum-free ham’s F12 media.

### Insulin receptor purification from 293T IR cells for maltoside detergent assay

293T IR cells (at 95% confluency) were harvested from eight 15 cm plates in PBS by pipetting. The cells from the plates were combined and centrifuged at 1000*g* for 5 min at 4 °C. Supernatant was removed and cell pellet was stored at −80 °C. For IR purification, the pellet was lysed at 4 °C with 40 ml purification lysis buffer (20 mM Tris pH 8.0, 400 mM NaCl, 10% (v/v) glycerol, 1% (v/v) Triton X-100, 1 mM EDTA, 5 μg/ml leupeptin, 5 μg/ml aprotinin) while mixing on an end-over-end rotor for 1 h. All the following steps were also at 4 °C unless otherwise noted. Lysate was then centrifuged at 12,520*g* for 30 min and the supernatant solution was filtered through a 0.8 μm syringe filter (Millex-AA, Millipore) before adding it to 2 ml of Strep-Tactin superflow resin (Qiagen), which was pre-equilibrated with 10 ml purification lysis buffer. Cleared lysate was incubated with resin for 30 min before pouring it into a 1.5 cm diameter glass chromatography column. The column was then rinsed with 30 column volumes (CV) of buffer A (20 mM Tris pH 8.0, 200 mM NaCl, 10% glycerol, 0.1% (v/v) Triton X-100). Beads in 50% slurry were transferred to a 50 ml polypropylene conical tube (Corning) and basal phosphorylation was removed by treatment with soluble glutathione s-transferase tagged *Yersinia* tyrosine phosphatase (YOP) (53) for 30 min at room temperature on an end-to-end rotor. YOP phosphatase was inhibited with 20 mM activated SOV (42) and incubated for an additional 10 min at room temperature. Beads were transferred back into the column and soluble YOP was washed away with 30 CV of buffer A. The detergent was then changed to DDM by washing the beads with 10 CV of Buffer B (20 mM Tris pH 8.0, 200 mM NaCl, 0.29 mM DDM). IR was eluted in buffer B containing 2.5 mM D-desthiobiotin. Purified IR was used without an additional concentration step.

### *In vitro* autophosphorylation in alkyl maltoside micelles

IR eluted in DDM was diluted 1:10 into solutions containing DM, DDM, TDM, or HDM at a concentration 1.25 times (0.5 + critical micelle concentration (CMC)) mM detergent in buffer C (20 mM Tris pH 8.0, 200 mM NaCl). For each detergent condition, 40 μl of IR in each alkyl maltoside detergent was added to 10 μl kinase reaction buffer (1 mM ATP, 1 mM MgCl_2_, 1 mM activated SOV in Tris pH 8) with or without 100 nM insulin to start the reaction and incubated for 5 min in a 23 °C water bath. The final detergent concentration was (0.5 + CMC) mM (*i.e.*, for DDM with CMC 0.17 mM, the final concentration was 0.5 mM + 0.17 mM = 0.67 mM). The reactions were stopped by adding 15 μl of 5× Laemmli buffer and analyzed by western blotting.

### *In vitro* radiometric kinase assay

IR in alkyl maltoside detergents was prepared as for autophosphorylation reactions to give (0.5 +CMC) mM final detergent concentration in kinase reactions. Reactions were started by adding 40 μl IR in alkyl maltoside micelles to 10 μl radioactive kinase reaction buffer (0.4 mM ATP, 1 μCi [γ-^32^P]-ATP (PerkinElmer, 10 mCi/ml, 25–50 cpm/mol), 1 mM MgCl_2_, 0.7 mM synthetic peptide KKEEEEYMMMMG (E_4_YM_4_), 1 mM SOV, and 1 mM BSA) with or without 100 nM insulin. The reactions were incubated in 30 °C water bath for 15 min and quenched by adding 18 μl cold 50% (v/v) trichloroacetic acid. Samples were centrifuged at 9296*g* for 2 min to pellet IR and 35 μl of supernatant was spotted on P81 phospho-cellulose paper (Whatman). Remaining [γ-^32^P]-ATP was removed by washing P81 papers in 0.5% (v/v) cold phosphoric acid thrice for 10 min each. Finally, P81 papers were dried, and radioactivity was measured in Hidex 300 SL scintillation counter.

### Lipid extraction from cells

For lipid exchange samples, cells were air dried on plate after PBS washes for 10 min in room temperature and then 1 ml of 3:2 (v:v) hexanes:isopropanol was added to each plate. The plates were kept on a rocker for 30 min and the organic solvent was transferred to a borosilicate glass test tube and stored in −20 °C. The remaining cell debris on plate was dissolved in 1 N NaOH and used for protein quantification by Bradford assay.

### HP-TLC of lipids

Extracted lipids were dried under N_2_ gas and then redissolved in 1:1 (v:v) chloroform:methanol. Aliquots were loaded on HP-TLC plates (HP-TLC Silica Gel 60 plates (Merck)). Protein quantification showed samples had similar amounts of lipid loaded per lane. Lipid samples were chromatographed in 65:35:5 (v:v:v) chloroform:methanol:28.0 to 30.0 (v/v) % ammonium hydroxide to separate phospholipids. For measuring lipid content, plates were sprayed with a solution of 3% (w/v) cupric acetate and 8% (v/v) phosphoric acid dissolved in water. The plates were air dried and charred at 180 to 200 °C to detect lipid bands. Band intensity was then measured using ImageJ program. For lipid exchange samples, exogenous lipid introduced was estimated from the decrease in cellular SM bands, as staining is near linear in SM concentration (56).

### Flow cytometry for detecting IR on plasma membrane

After lipid exchange as described above, cells were dislodged from plates using enzyme-free cell dissociation solution (Millipore), spun down at 300*g*, 4 °C for 5 min, and washed with blocking buffer (0.5% BSA, 2 mM EDTA in PBS) at room temperature. Manufacturer’s protocol was followed for fluorescent antibody staining. Cells were resuspended in 100 μl blocking buffer, CD220-PE antibodies against IR alpha chain or recombinant antibody (REA) control (S)-PE (Miltenyi Biotech) were added, and the samples were incubated at 4 °C in the dark for 10 min. Samples were washed at room temperature in PBS blocking buffer again. Flow cytometry analysis was performed using FACSCalibur (BD Bioscience) collecting 10,000 events per sample. Data was analyzed using FlowJo version 10 software.

### Cell viability assessment using flow cytometry

After lipid exchange and an additional 1 h recovery step in complete media in a 37 °C, 5% CO_2_ incubator, cell viability was assessed using propidium iodide (Thermo Fisher) as described previously (29). Briefly, after lipid exchange, cells were washed in PBS thrice and 1 ml complete media was added. The cells were then incubated in a 37 °C, 5% CO_2_ incubator for 1 h, which was followed by a PBS wash. The cells were then detached using trypsin, washed in PBS, and resuspended in 100 μl of binding buffer (50 mM HEPES pH 7.4, 700 mM NaCl, 12.6 mM CaCl_2_) per million cells. Propidium iodide was added (1 μg/ml) and samples were incubated at room temperature while being protected from light for 15 min on a shaker. Samples were diluted with 400 μl binding buffer and analyzed using FACSCalibur (BD Biosciences) flow cytometer collecting 50,000 events per sample.

### Quantification of western blot images and ensuring linearity of detection

Western blot bands were quantified using ImageJ software analysis. Individual pYpY IR autophosphorylation bands were normalized to total IR-β bands for corresponding samples. To confirm that the signal from antibodies is linear in concentration at the exposure used, a standard curve of dilutions from one sample (usually untreated sample or sample with the highest intensity) was run on the same blot as the experiments. Averages and standard deviations from independent experiments were then calculated.

### Statistical analysis and replicates

When desired, Student’s *t* test (unpaired, two-tailed *t* test with equal variances) was calculated to determine significant differences between mean values.

## Data availability

All relevant data are contained within this article and in the supporting information.

## Supporting information

This article contains supporting information (35, 37, 57, 58, 59, 60, 61).

## Conflict of interest

The authors declare that they have no conflicts of interest with the contents of this article.
